# Efficacy of Rajahpravartini Vati in Adolescent Girls and Young Women With Primary Dysmenorrhea: Protocol for a Prospective, Double-Blind, Randomized, Placebo-Controlled Clinical Trial

**DOI:** 10.2196/101456

**Published:** 2026-10-22

**Authors:** Arvind Kumar, Gurucharan Bhuyan, Vijaya Lekshmi R, BCS Rao, Prashant Shinde, Arunabh Tripathi, Narayanam Srikanth, Rabinarayan Acharya

**Affiliations:** 1 CCRAS-Regional Ayurveda Research Institute, Itanagar, Arunachal Pradesh, 791111, India, 91 9660033435; 2 Central Council for Research in Ayurvedic Sciences, New Delhi, India

**Keywords:** Rajahpravartini Vati, primary dysmenorrhea, ayurveda, randomized controlled trial, menstrual pain, adolescent health, placebo-controlled trial

## Abstract

**Background:**

Primary dysmenorrhea is a prevalent menstrual disorder affecting adolescents and young women worldwide and is associated with substantial impairment in daily functioning, academic performance, psychosocial well-being, and quality of life. Conventional treatments, including nonsteroidal anti-inflammatory and hormonal therapies, often provide temporary symptomatic relief but may cause adverse effects with prolonged use. Rajahpravartini Vati, a classical Ayurvedic herbomineral formulation traditionally indicated for painful menstruation, has shown preliminary therapeutic potential but lacks evaluation in a rigorous placebo-controlled clinical trial.

**Objective:**

This study aims to evaluate the efficacy of Rajahpravartini Vati in adolescent girls and young women aged 12 to 24 years with primary dysmenorrhea.

**Methods:**

This study protocol describes a prospective, double-blind, randomized, placebo-controlled, parallel-group clinical trial to be conducted at the Regional Ayurveda Research Institute, Itanagar, Arunachal Pradesh, India. A total of 100 eligible participants will be randomized in a 1:1 ratio to receive either Rajahpravartini Vati (500 mg twice daily) or a matched placebo for 3 consecutive menstrual cycles. Treatment will begin 3 days before the expected onset of menstruation and continue until 3 days after onset, followed by 1 observational cycle. The primary outcome is the change in dysmenorrhea severity measured using the Working Ability, Location, Intensity, Days of Pain, Dysmenorrhea score. Secondary outcomes include pain intensity and duration, rescue medication use, psychosomatic symptoms, and perceived stress. Both intention-to-treat and per-protocol analyses are planned.

**Results:**

The study was sanctioned in July 2025, and the first installment of funds was released in September 2025. Ethics approval was obtained on July 16, 2025 (IEC-RARI/2025-26/02), and the trial was prospectively registered with the Clinical Trial Registry–India (CTRI/2025/07/091892) on July 28, 2025. Participant recruitment began in June 2026, and is ongoing. To date, 15 participants had been screened, 6 enrolled, and 9 were classified as screen failures. Data analysis has not yet commenced, and results are expected to be available for publication in 2028.

**Conclusions:**

This trial is expected to generate evidence regarding the efficacy of Rajahpravartini Vati in managing primary dysmenorrhea among adolescent girls and young women and may help inform future integrative approaches to menstrual health management.

## Introduction

### Background

Primary dysmenorrhea (PD) is one of the most prevalent gynecological conditions affecting menstruating women worldwide and represents a significant public health concern. Global estimates indicate a prevalence ranging from 16.8% to 81%, with particularly high rates among adolescents and young adults; studies have reported a prevalence of 67% to 90% in women aged 17 to 24 years and up to 93% among teenagers experiencing menstrual pain [1-5]. In India, reported prevalence ranges between 50% and 87.8%, reflecting a similarly widespread burden [6]. Established risk factors include early menarche, nulliparity, smoking, and family history [7,8].

The pathophysiology is largely attributed to elevated prostaglandin F2α and E2 levels, which increase myometrial contractions, induce vasoconstriction, and sensitize pain pathways [9]. Clinically, PD manifests as cramping lower abdominal pain that may radiate to the back and thighs and is often accompanied by nausea, vomiting, diarrhea, fatigue, and headache [9-14]. Beyond physical discomfort, PD substantially affects school attendance, work productivity, and psychosocial well-being, contributing to measurable declines in quality of life [15-19].

Despite its high prevalence and functional impact, current therapeutic approaches remain primarily symptomatic. Conventional management relies on nonsteroidal anti-inflammatory drugs, acetaminophen, and hormonal contraceptives [9]. Although these therapies are effective for many individuals, they are not curative, may be contraindicated in some patients, and can produce adverse effects with repeated or prolonged use. These limitations have stimulated interest in alternative or complementary strategies that may provide effective symptom control with improved tolerability.

### Rationale

Ayurvedic medicine describes Rajahpravartini Vati (RPV) as a classical herbomineral formulation traditionally indicated for painful or delayed menstruation. It contains purified borax, *Ferula assa-foetida* exudate, ferrous sulfate, and *Aloe barbadensis* extract, whose constituents have been associated with anti-inflammatory, analgesic, and uterotonic actions in pharmacological literature [20-22]. Searches of biomedical and traditional medicine databases, including PubMed, the Ayush Research Portal, the Digital Helpline for Ayurveda Research Articles, and the Clinical Trial Registry–India, identified 8 completed and 1 ongoing study evaluating RPV in PD, either alone or in combination with other Ayurvedic formulations [23-31]. However, most available studies are single-arm or small postgraduate investigations and demonstrate methodological limitations such as inadequate sample sizes, absence of appropriate control groups, lack of randomization or blinding, and limited peer-reviewed dissemination. These weaknesses prevent firm conclusions regarding efficacy and restrict integration into evidence-based clinical practice.

A rigorously designed placebo-controlled trial is therefore necessary to evaluate RPV under contemporary clinical research standards. Adolescents and young women represent the population most affected by PD and frequently seek treatment options perceived as safer and culturally acceptable. Generating reliable evidence in this group is important for informing integrative approaches to menstrual health and for guiding future research and policy development. This study is positioned to address a critical evidence gap and to provide a methodological framework for evaluating traditional medicine interventions within modern clinical trial systems.

### Objectives

The primary objective of this study is to evaluate the efficacy of RPV compared with placebo in reducing the severity of PD in adolescent girls and young women. Secondary objectives include assessing its effects on pain duration and intensity, frequency of rescue medication use, psychosomatic symptoms, and perceived stress. Validated outcome instruments will be used, including the Working ability, Location, Intensity, Days of pain, Dysmenorrhea (WaLIDD) score, visual analog scale (VAS), Moos Menstrual Distress Questionnaire (MDQ), and the 10-item Perceived Stress Scale (PSS-10). Through a randomized, double-blind, placebo-controlled design, the study aims to generate robust clinical evidence regarding the therapeutic role of this classical Ayurvedic formulation in the management of PD.

## Methods

### Study Design and Setting

The proposed clinical trial uses a randomized, double-blind, placebo-controlled, parallel-group design with a 1:1 allocation ratio. This phase 3 interventional trial follows an efficacy framework. This study protocol has been prepared in accordance with the SPIRIT (Standard Protocol Items: Recommendations for Interventional Trials) (Checklist 1) 2025 statement. All logistical preparations, including drug procurement, blinding, laboratory arrangements, assessment tool licensing, and case report form printing, have been completed.

The trial will be conducted at the outpatient department of Regional Ayurveda Research Institute (RARI), Itanagar, Arunachal Pradesh, India. The study duration spans 27 months, including a 3-month preparatory period; 15 months for screening, recruitment, intervention, and follow-up; 3 months for statistical analysis; and 6 months for publication and technical report preparation.

### Study Participants

Eligible participants will be menstruating female individuals aged 12 to 24 years who are nulliparous and have completed at least 1 year since menarche. Participants reporting a history of menstruation-associated pain for at least 3 of the last 6 menstrual cycles and demonstrating regular menstrual cycles (≤9 days of variation between shortest and longest cycles) lasting 24 to 38 days with bleeding duration ≤8 days and normal flow during the last 3 cycles will be included. Written informed assent and/or consent for study participation over the subsequent 5 months will be collected from the participants before screening.

Participants will be excluded if they have a known or previously diagnosed gynecological or pelvic pathology or clinical features suggestive of such pathology, including uterine fibroids, gynecological malignancy, female hormonal disorders, polycystic ovarian disease, endometriosis, pelvic inflammatory disease, metrorrhagia, menorrhagia, leucorrhea, or previous gynecological surgery within the past 6 months. Other exclusion criteria include systemic disorders likely to affect menstrual physiology such as thyroid dysfunction, mental disorders, and moderate anemia (hemoglobin <10 gm/dL), as well as hypertension, diabetes mellitus, hepatic impairment (serum glutamic oxaloacetic transaminase [SGOT], serum glutamic pyruvic transaminase [SGPT], and total bilirubin >2 times upper normal limits), and renal impairment (serum creatinine above normal limits).

Participants will also be excluded for persistent pelvic pain throughout the menstrual cycle; concurrent neurological, pulmonary, endocrine, or unstable cardiovascular disease; current or recent use of intrauterine devices or oral contraceptive pills during the last 3 cycles; plans to conceive within 6 months; or ongoing prolonged medication for other medical conditions.

Eligibility will be determined through a detailed medical and menstrual history, clinical examination and assessment, and specified laboratory investigations. These comprehensive assessments, together with the specified inclusion and exclusion criteria, will be used to identify features suggestive of secondary dysmenorrhea. Routine pelvic imaging is not mandated for screening purposes.

### Study Interventions

The active test intervention consists of RPV tablets, each containing 250 mg of the formulation as described in classical Ayurvedic therapeutics, the Ayurvedic Pharmacopoeia of India, and the Ayurvedic Formulary of India. RPV is a herbomineral formulation comprising equal proportions of Shuddha Tankana (purified borax), Shuddha Hingu (purified exudate of *Ferula assa-foetida*), Shuddha Kasisa (purified ferrous sulfate), and Kanyasara (solid dried leaf extract of *Aloe barbadensis*), triturated with Kumari Swarasa (*Aloe barbadensis* leaf juice). Participants will receive 500 mg (2 tablets) twice daily with lukewarm water from 3 days prior to the expected onset of the menstrual cycle until 3 days after the onset of bleeding, administered for 3 consecutive menstrual cycles.

The control intervention consists of placebo tablets, containing dicalcium phosphate, microcrystalline cellulose, and starch, manufactured to match the active test intervention in appearance, weight, and packaging. Participants will receive an identical dosing regimen of 500 mg (2 tablets) twice daily with lukewarm water following the same administration schedule as the active intervention. The placebo formulation used in the study will adhere to the Indian Pharmacopoeial standards.

The investigational products were procured from the Indian Medicines Pharmaceutical Corporation Limited (IMPCL), Mohan, Uttarakhand. The raw materials used in the formulations underwent quality control testing against the applicable pharmacopoeial standards. The finished products were also subjected to physicochemical and analytical quality testing, including testing for heavy metals, aflatoxins, pesticide residues, and microbiological parameters, to ensure conformity with the specified quality requirements. The supplied batch was accompanied by a certificate of analysis confirming compliance with the specified quality standards. The formulations are packed and sealed in airtight containers and stored under controlled conditions at the study site.

### Rescue Medication

A designated modern medicine physician will prescribe a fixed-dose combination comprising 250 mg mefenamic acid and 10 mg of dicyclomine to all the participants as a rescue medication. The rescue medication will be procured from a good manufacturing practice–certified, reputed, and licensed manufacturer (following Indian Pharmacopoeial standards) in the local market. The modern medicine physician and investigators will issue the rescue medicine along with the trial drug to the participants, providing thorough consultation and instructions regarding the indication, dosage, frequency, duration, and administration of the rescue medicine.

### Concomitant Care

During the study period, participants may receive concomitant care or medications as clinically necessary that are not expected to interfere with the outcomes of the trial. However, the use of any medications or therapies that may influence menstrual physiology or the perception of pain, other than the prescribed rescue medication, as well as Ayurvedic formulations with similar indications, will not be permitted during the trial period. All concomitant medications taken by the participants will be recorded in the case report form (CRF) with details, including drug name, dose, frequency, and duration. The investigator will assess and document any potential impact of such therapies on the study outcomes. Participants will be instructed to inform the study team before initiating any new treatment during the trial.

### Outcome Measures

The primary outcome measure is the change in the severity of PD assessed using the WaLIDD score [32]. The primary efficacy comparison will be the between-group difference in the change in the WaLIDD score from baseline to the end of the third menstrual cycle, corresponding to the completion of the 3-cycle intervention period. WaLIDD will also be assessed after the first, second, and fourth menstrual cycles, with the fourth-cycle assessment representing the treatment-free follow-up period and used to evaluate persistence of the treatment effect after discontinuation of the intervention. Secondary outcome measures include changes in pain duration and intensity measured using VAS scores recorded every 12 hours during menstrual periods at baseline and during cycles 1 to 4, frequency of rescue medication use at the end of cycle 4, psychosomatic status assessed using the Moos MDQ at baseline and cycles 3 to 4, and perceived stress levels evaluated using the PSS-10 at baseline and cycles 3 to 4 [33,34]. Permission and licensing for the use of the Moos MDQ have been obtained from the copyright holder.

### Safety Monitoring

Participants will be monitored for adverse events (AEs) and suspected adverse drug reactions (ADRs) throughout the study period. At each follow-up visit, participants will be assessed for the occurrence of any AE or ADR, and any such event will be documented in the CRF, including its nature, severity, duration, management, outcome, and suspected relationship to the study intervention, as applicable. Appropriate medical management will be provided when required.

The allocated intervention may be discontinued for individual participants under specific circumstances to ensure participant safety and protocol integrity. These include voluntary withdrawal by the participant at any stage of the study, development of AEs or suspected ADRs related to the study medication, clinical deterioration necessitating emergency care or hospitalization, or the emergence of any condition listed in the exclusion criteria during the trial period. Additionally, noncompliance with the study protocol, such as failure to adhere to the dosing schedule or attend follow-up visits, may also warrant discontinuation. If a participant becomes pregnant during the course of the study, the intervention will be immediately stopped due to potential safety concerns. The decision to discontinue the intervention will be made at the discretion of the investigator, who will document a detailed justification and ensure appropriate follow-up and medical management. All such cases will be recorded in the CRF and reported to the Institutional Ethics Committee (IEC) and the sponsor in accordance with applicable regulatory requirements.

### Sample Size

The study will recruit 100 participants (50 per group) based on power calculations. Sample size determination considered an expected mean WaLIDD score of 7 (SD 2.4) at baseline, with anticipated changes of 30% (2.1 points) following the intervention and 10% (0.7 points) following placebo. Using a 95% CI and 80% power, the calculated sample size was 46 participants per group. After accounting for a 5% dropout rate, the required sample size increased to 48 per group, which was rounded up to 50 participants per group for a total of 100 participants.

### Recruitment and Retention Strategies

Participant recruitment will be facilitated through menstrual health awareness campaigns at local high schools, colleges, coaching centers, and offices. Potential participants and their parents (for participants <18 years) will receive appropriate counseling and educational materials on menstrual health. Retention strategies include the provision of conveyance allowances, telephonic reminders, and counseling about potential research benefits.

### Randomization, Allocation Concealment, and Blinding

Participants will be randomized using a computer-generated random number sequence with a 1:1 allocation to the intervention and control groups. Double blinding is ensured through identical packaging and appearance of the active and placebo interventions. The blinding and labeling process has been completed by centrally designated personnel independent of outcome assessment, and the allocation code is securely preserved by the statistical unit of the Central Council for Research in Ayurvedic Sciences (CCRAS) headquarters and will remain concealed until the completion of the study and final data analysis.

### Data Collection

Following the acquisition of written consent or assent, potential participants visiting RARI’s outpatient department with menstrual pain complaints will undergo eligibility screening through clinical assessment and laboratory investigations, including serum SGOT; SGPT; total, direct, and indirect bilirubin; creatinine; fasting blood sugar; serum thyroid-stimulating hormone, and hemoglobin concentration (eg, g/dL). A certified external laboratory has been identified and contracted for all protocol-specified investigations to ensure standardized and timely laboratory assessments. Eligible participants will be enrolled in the study and will receive VAS leaflets to record pain scores during their upcoming menstrual cycle.

Enrolled participants will complete comprehensive baseline assessments using standardized questionnaires. Previously issued VAS leaflets will be collected, trial medications will be dispensed based on expected menstrual onset dates, and rescue medication will be provided with detailed use instructions. Participants will receive drug compliance report forms (DCRFs) and menstrual diaries for recording relevant trial events. Conveyance charges will be provided for each visit.

During visits following the completion of consecutive menstrual cycles 1 to 3, participants will return unused medications and completed compliance forms. Reassessments will be conducted using protocol-specified tools, with subsequent medication dispensing and diary collection. Completion of cycle 3 marks the final intervention assessment without additional medication dispensing (Figure 1).

The final assessment occurs after completion of the fourth menstrual cycle, with comprehensive evaluation using all protocol-specified instruments and final menstrual diary collection (Table 1).

**Figure 1.**
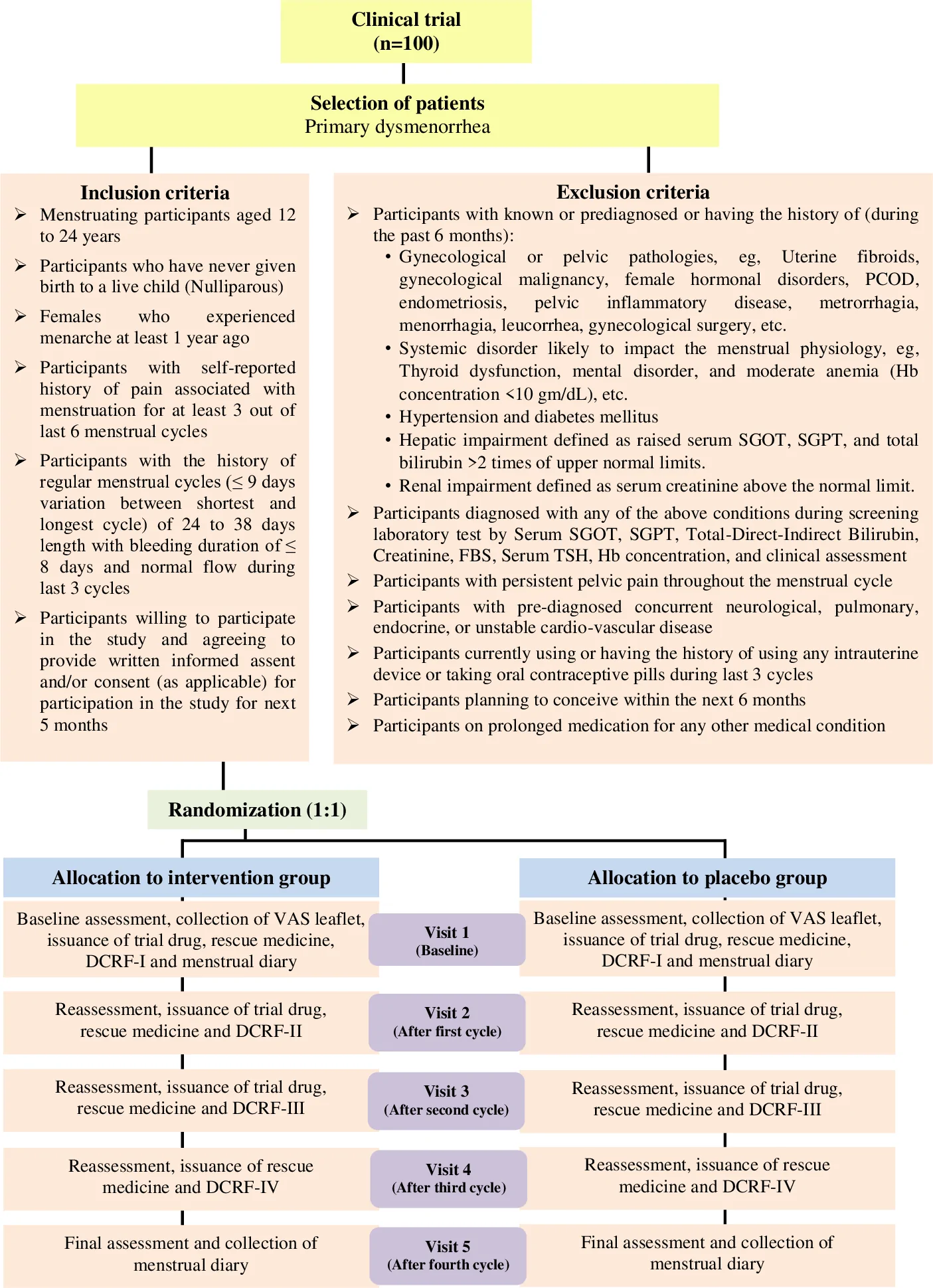
Flow diagram of participant screening, randomization, intervention, and follow-up in the double-blind, randomized, placebo-controlled trial of Rajahpravartini Vati for primary dysmenorrhea among participants aged 12 to 24 years, where participants receive the intervention for 3 consecutive menstrual cycles, followed by 1 treatment-free observational cycle. DCRF: drug compliance report form; FBS: fasting blood sugar; Hb: hemoglobin; PCOD: polycystic ovarian disease; SGOT: serum glutamic oxaloacetic transaminase; SGPT: serum glutamic pyruvic transaminase; TSH: thyroid-stimulating hormone; VAS: visual analog scale.

**Table 1.** Schedule of screening, enrollment, intervention, outcome assessment, safety monitoring, and follow-up in the double-blind, randomized, placebo-controlled trial of Rajahpravartini Vati for primary dysmenorrhea among participants aged 12 to 24 years^a^.

Procedure	Screening	Baseline	Cycle 1	Cycle 2	Cycle 3	Follow-up
Consent or assent sign	✓					
Eligibility evaluation	✓					
Laboratory tests	✓					
Issuance of visual analog scale leaflet	✓					
Demographics and clinical assessment		✓				
WaLIDD^b^ scoring		✓	✓	✓	✓	✓
Moos Menstrual Distress Questionnaire scoring		✓			✓	✓
10-item Perceived Stress Scale scoring		✓			✓	✓
Issuance of trial drug pack		✓	✓	✓		
Issuance of rescue medicine		✓	✓	✓		
Issuance of DCRF^c^		✓	✓	✓		
Issuance of menstrual diary		✓				
Collection of remaining trial drug			✓	✓	✓	
Collection of remaining rescue medicine			✓	✓	✓	
Collection of filled DCRF			✓	✓	✓	
Examination of menstrual diary			✓	✓	✓	
Collection of menstrual diary						✓
Payment of conveyance charge		✓	✓	✓	✓	✓
Recording of rescue medication			✓	✓	✓	✓
Recording of concomitant therapy			✓	✓	✓	✓
Recording of AE^d^, SAE^e^, and ADR^f^			✓	✓	✓	✓
Recording of menstrual history		✓	✓	✓	✓	✓

a The intervention is administered for 3 consecutive menstrual cycles, followed by 1 treatment-free follow-up cycle.

b WaLIDD: Working ability, Location, Intensity, Days of pain, Dysmenorrhea.

c DCRF: drug compliance report form.

d AE: adverse event.

e SAE: serious adverse event.

f ADR: adverse drug reaction.

### Data Management

All data for this study will be collected prospectively using a designated CRF. At each scheduled visit, demographic, clinical, laboratory, and outcome-related data will be documented. Participants will also maintain a menstrual diary to record real-time events such as the onset and cessation of menstruation and pain, VAS scores (12-hourly), timing and use of the rescue medication, and any AEs. The menstrual diaries will be reviewed by the investigators during each follow-up visit for completeness and accuracy. The CRF data will be entered into a secure, password-protected e-CRF. The CRFs will be maintained by the principal investigator. Personal identifiers will be coded to ensure confidentiality, and access to raw data will be restricted to authorized personnel only. Any changes to the recorded data will be traceable through an audit trail. Data cleaning and validation will be done prior to statistical analysis, and final datasets will be archived as per the CCRAS policies. The data will be shared with the sponsor and the IEC, as required.

### Statistical Analysis

The data will be checked for accuracy and completeness. Missing data will be imputed using a regression imputation approach. Quantitative variables, including WaLIDD and VAS scores, will be summarized as mean (SD) or median (IQR), as appropriate. Between-group comparisons will be performed using the independent-samples 2-tailed *t* test or Mann-Whitney *U* test, depending on the distribution of the data. Within-group comparisons across time points will be performed using paired *t* tests or repeated measures ANOVA, as appropriate. For nonnormally distributed data, the Wilcoxon signed-rank test or Friedman test will be used, as applicable. Categorical variables will be summarized as frequencies and percentages and analyzed using the chi-square test or Fisher exact test, as appropriate. Within-group comparisons of categorical variables across time points will be performed using the Cochran Q test.

The primary longitudinal analysis will be performed using generalized estimating equations (GEE) to account for repeated measurements within participants. The GEE model will evaluate the effects of treatment group, time, and the treatment group×time interaction, with corresponding *P* values reported. Other relevant covariates or factors influencing the outcomes may also be considered in the model, as appropriate.

The intention-to-treat (ITT) population will include all randomized participants, irrespective of subsequent dropout or withdrawal, and participants will be analyzed according to their originally assigned intervention group. Missing data in the ITT analysis will be handled using the prespecified regression imputation approach. A per-protocol analysis will also be performed to evaluate the robustness of the findings. Safety analyses will include descriptive summaries of AEs, ADRs, and serious adverse events, stratified by intervention group. One interim analysis will be conducted after completion of all follow-up assessments for 50% of the participants. A 2-sided *P*<.05 will be considered statistically significant. Statistical analyses will be performed using SPSS (version 28.0; IBM Corp).

### Study Monitoring and Auditing

Study conduct will be monitored by a monitoring committee constituted by CCRAS headquarters, New Delhi, ensuring adherence to protocol requirements and good clinical practice standards throughout the trial duration. The team will also conduct periodic on-site audits.

### Ethical Considerations

The study was approved by the IEC of RARI, Itanagar, Arunachal Pradesh (IEC-RARI/2025-26/02) on July 16, 2025. Written informed assent will be obtained from participants aged <18 years along with parental consent, while participants ≥18 years will provide written informed consent. All enrolled participants will be covered under a clinical trial insurance policy applicable throughout the duration of the study, which will include provisions for medical management in the unlikely event of any trial-related harm. Participant confidentiality will be maintained throughout the study, and necessary permissions for questionnaire use will be obtained where applicable. The trial has been prospectively registered with the Clinical Trial Registry–India (CTRI/2025/07/091892) on July 28, 2025.

## Results

The study was sanctioned in July 2025, and the first installment of funds was released in September 2025. Ethics approval was obtained on July 16, 2025 (IEC-RARI/2025-26/02), and the trial was prospectively registered with the Clinical Trial Registry–India (CTRI/2025/07/091892) on July 28, 2025. Participant recruitment commenced in June 2026 and is ongoing. To date, 15 participants had been screened, of whom 6 were enrolled and 9 were classified as screen failures. One enrolled participant subsequently dropped out. Recruitment and data collection are ongoing, and data analysis has not yet commenced. Based on the planned study timeline, the results are expected to be available for publication in 2028.

## Discussion

### Novel Aspects of the Study

The protocol describes a rigorously designed, randomized, double-blind, placebo-controlled clinical trial evaluating the efficacy of RPV in adolescent girls and young women with PD. The study addresses an important evidence gap in the evaluation of Ayurvedic interventions for menstrual health using validated outcome measures and contemporary clinical trial methodology. The use of standardized recruitment procedures, validated assessment instruments, provision of a comparator group, allocation concealment, double blinding, ITT and per-protocol analyses is expected to improve methodological rigor and interpretability.

If demonstrated to be effective and safe, RPV may provide an additional nonhormonal and culturally acceptable management option for adolescents and young women experiencing PD.

### Challenges, Limitations, and Strengths

Recruitment and retention of adolescent female participants constitute a significant operational challenge in menstrual health research. Cultural sensitivity around menstruation and hesitancy to discuss menstrual pain openly can lead to underreporting and recruitment delays. To address this, the study incorporates structured awareness sessions in nearby schools and colleges, parental counseling for minors, and reimbursement of travel expenses—strategies known to improve participation and compliance in adolescent cohorts [9,17].

Ensuring drug adherence and proper maintenance of menstrual diaries are additional challenges in multivisit trials. To mitigate this, the present trial uses simplified diary formats, periodic telephonic reminders, and provision of DCRFs.

Provision of rescue medication following prescription and appropriate instructions from a designated modern medicine physician not only ensures ethical management of acute pain but also facilitates objective recording of analgesic use as a secondary outcome.

Blinding and placebo formulation posed methodological complexities due to the organoleptic characteristics of Ayurvedic formulations. Achieving indistinguishable placebo tablets in terms of color, taste, and weight required iterative development with the IMPCL, ensuring adherence to the Ayurvedic Pharmacopoeia of India, Ayurvedic Formulary of India, and Indian Pharmacopoeia standards. Such stringent matching enhances the reliability of blinding, which has been a common shortcoming in earlier Ayurvedic trials [23-31].

The use of validated tools, such as the WaLIDD score, Moos MDQ, and PSS-10, ensures a multidimensional assessment encompassing the physical, psychological, and functional domains of PD [32-34].

Another methodological strength lies in the integration of both ITT and per-protocol analyses, enhancing interpretability and reproducibility. The sample size was determined statistically to ensure adequate power, addressing the limitation of small sample studies previously reported for RPV. The inclusion of adolescent and young adult female participants aged 12 to 24 years also strengthens external validity by focusing on the population most affected by PD [3,5,15].

From a translational standpoint, this trial represents a crucial step toward evidence-based validation of traditional formulations. If RPV demonstrates significant efficacy and safety over placebo, it could provide a cost-effective, culturally acceptable, and nonhormonal therapeutic alternative for PD, aligning with the global call for integrative approaches in menstrual health management [18,19].

Limitations include the study’s single-center design, which may limit generalizability across diverse ethnic populations. Additionally, long-term efficacy and recurrence beyond the follow-up period will also not be evaluated.

The use of a standardized formulation and fixed treatment regimen is necessary to maintain intervention consistency, blinding, and reproducibility in this randomized controlled trial. However, this approach may not fully reflect individualized Ayurvedic clinical practice, in which therapeutic decisions may be tailored according to individual patient characteristics and clinical presentation. Therefore, the findings of this study will primarily reflect the efficacy of the standardized RPV regimen evaluated in this trial rather than the effectiveness of individualized Ayurvedic management of PD.

### Expected Outcomes

Overall, this trial is expected to generate high-quality clinical evidence addressing a significant gap in women’s health research, while also offering methodological insights for conducting placebo-controlled Ayurvedic trials in real-world conditions.

## Supplementary material

10.2196/101456 Checklist 1 SPIRIT 2025 checklist.

## Abbreviations

ADR: adverse drug reaction
AE: adverse event
CCRAS: Central Council for Research in Ayurvedic Sciences
CRF: case report form
CTRI: Clinical Trial Registry–India
DCRF: Drug Compliance Report Form
GEE: Generalized Estimating Equation
IEC: Institutional Ethics Committee
IMPCL: Indian Medicines Pharmaceutical Corporation Limited
ITT: intention-to-treat
MDQ: Menstrual Distress Questionnaire
PD: primary dysmenorrhea
PSS-10: 10-Item Perceived Stress Scale
RARI: Regional Ayurveda Research Institute
RPV: Rajahpravartini Vati
SGOT: serum glutamic oxaloacetic transaminase
SGPT: serum glutamic pyruvic transaminase
SPIRIT: Standard Protocol Items: Recommendations for Interventional Trials
VAS: visual analog scale
WaLIDD: Working ability, Location, Intensity, Days of pain, Dysmenorrhea
